# A Position- and Similarity-Aware Named Entity Recognition Model for Power Equipment Maintenance Work Orders

**DOI:** 10.3390/s25072062

**Published:** 2025-03-26

**Authors:** Ziming Wei, Shaocheng Qu, Li Zhao, Qianqian Shi, Chen Zhang

**Affiliations:** College of Physical Science and Technology, Central China Normal University, Wuhan 430079, China; zim_w@mails.ccnu.edu.cn (Z.W.); zhli@mails.ccnu.edu.cn (L.Z.); shi5210@mails.ccnu.edu.cn (Q.S.); m13403053751@mails.ccnu.edu.cn (C.Z.)

**Keywords:** named entity recognition, power equipment maintenance, transformer, artificial intelligence

## Abstract

Power equipment maintenance work orders are vital in power equipment management because they contain detailed information such as equipment specifications, defect reports, and specific maintenance activities. However, due to limited research into automated information extraction, valuable operational and maintenance data remain underutilized. A key challenge is recognizing unstructured Chinese maintenance texts filled with specialized and abbreviated terms unique to the power sector. Existing named entity recognition (NER) solutions often fail to effectively manage these complexities. To tackle this, this paper proposes a NER model tailored to power equipment maintenance work orders. First, a dataset called power equipment maintenance work orders (PE-MWO) is constructed, which covers seven entity categories. Next, a novel position- and similarity-aware attention module is proposed, where an innovative position embedding method and attention score calculation are designed to improve the model’s contextual understanding while keeping computational costs low. Further, with this module as the main body, combined with the BERT-wwm-ext and conditional random field (CRF) modules, an efficient NER model is jointly constructed. Finally, validated on the PE-MWO and five public datasets, our model shows high accuracy in recognizing power sector entities, outperforming comparative models on public datasets.

## 1. Introduction

**Figure 1:**
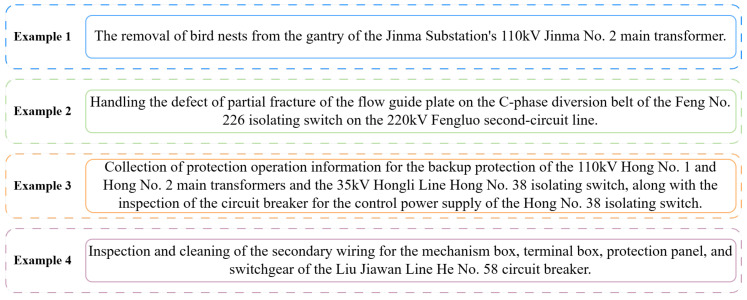
Examples of power equipment maintenance work orders (translated from original Chinese records).

As power companies rapidly develop, the scale of power equipment has expanded quickly, producing vast, diverse, and complex data during operation. Specifically, as illustrated in Figure 1, power equipment maintenance work orders include essential information such as equipment names, associated lines, damaged components, and maintenance statuses. For example, these details may involve tasks like the removal of bird nests from the gantry of a transformer (Example 1), the inspection of fractured components in equipment like isolating switches (Example 2), the collection of protection operation information for transformers and circuit breakers (Example 3), or maintenance operations on circuit breakers (Example 4). These work orders are often derived from a combination of sensor measurements, which provide real-time data on equipment performance (e.g., temperature, pressure, and voltage readings), and human experts’ observations, such as visual inspections and operational assessments. The sensor data serve as a critical source for detecting anomalies or performance degradation, which then triggers maintenance actions, as reflected in the work orders. For example, abnormal sensor readings may prompt the identification of issues such as a failing component or the need for cleaning, as seen in the examples from Figure 1, which include the removal of bird nests from a transformer or the inspection of an isolating switch. These maintenance work orders, when combined with sensor data, can form the basis for predictive maintenance models and enhance the development of smart grids [1,2]. However, the unstructured text format of these work orders prevents seamless integration with sensor data, creating challenges in performing comprehensive equipment monitoring. This lack of structure not only limits real-time data analysis but also makes it difficult to leverage the full potential of both sensor data and maintenance records in predictive maintenance and system optimization. Therefore, there is an urgent need for an efficient method to automatically extract valuable text information from power equipment maintenance work orders so as to enable better data fusion and analysis.

Named entity recognition (NER) is a subtask of natural language processing (NLP) that aims to locate and classify named entities in text into specific categories [3]. It is a crucial preliminary task for constructing knowledge graphs and knowledge question-answering systems [4,5]. With the advancement of deep neural networks, deep learning methods have replaced rule-based and dictionary-based approaches as the primary means of achieving NER. Collobert et al. [6] proposed a CNN-based NER method, while Huang et al. [7] introduced the BiLSTM-CRF model, which has become a classic model for subsequent NER tasks. In practical engineering fields, Li et al. [8] proposed a dictionary-enhanced machine reading comprehension method for NER, used to identify entity categories from bridge inspection texts. Liu et al. [9] employed BERT and BiLSTM algorithms to construct a model for NER in high-speed railway fault texts.

However, research on NER within the domain of electric power is sparse, particularly in the context of the Chinese language. Compared to English, Chinese presents challenges due to its ambiguous word boundaries and the prevalence of polysemous and homonymous terms. This complexity is further compounded in power equipment maintenance work orders, which contain numerous specialized and abbreviated terminologies. For instance, “Jinma Substation No. 508 disconnect switch” is typically abbreviated as “Jin 508 switch”. Furthermore, numerical entities in work orders require contextual information for accurate categorization, such as voltage levels (110 kV), temporal references (2020-0306), and equipment identifiers (the numerical 508 in “Jin 508 switch” denoting switch number). Additionally, the varying writing styles of different personnel further exacerbate the difficulty of entity recognition. This complexity poses significant challenges for integrating maintenance information with sensor data and achieving comprehensive equipment monitoring. Existing NER methods struggle to cope with the complex vocabulary and contexts in the electric power domain.

To address the aforementioned challenges, we propose a Chinese NER model designed to accurately identify entities within power equipment maintenance work orders. Moreover, our model outperforms other comparative models across five public datasets.

The main contributions of this paper are as follows:

- We construct the power equipment maintenance work orders (PE-MWO) dataset. The dataset comprises seven entity categories, encompassing a total of 7415 sentences and 238,869 characters.
- We develop a position- and similarity-aware attention module that refines the position embedding and attention score computation processes of the original Transformer model. Additionally, this module incorporates vector angle calculations to enhance the model’s capacity to detect token similarity information. This modification enables more nuanced contextual understanding in natural language processing tasks.
- Extensive experimental results provide compelling evidence for the efficacy of our proposed model. It demonstrates high recognition accuracy across both the PE-MWO dataset and five public datasets. This approach significantly advances the resolution of power industry-specific NER challenges, offering a robust reference for analogous domain-specific research.

## 2. Related Work

### 2.1. AI Applications in Power and Energy Management

Artificial intelligence technology is increasingly being applied in a wide range of research directions [10,11,12,13,14,15]. Recent advancements in AI have significantly enhanced power and energy system optimization and anomaly detection [16,17]. Wen et al. [18] developed a heterogeneous federated learning framework with CKKS homomorphic encryption and a CNN-LSTM hybrid model to address the class imbalance problem in electricity theft detection while preserving data privacy in smart grids. Peng et al. [19] introduced a feature extraction framework that reveals implicit energy behavior correlations among users, improving user-level demand forecasting accuracy by 14% and identifying energy waste patterns caused by behavioral inertia through a visual analytical interface. Razzak et al. [20] proposed a deep Q-learning-based framework combining multi-objective linear programming (MOLP) with four weighted and regressive moving average forecasting methods to balance consumer QoE and cost in smart grids, achieving superior performance in both metrics through iterative error reduction and intelligent stored power management. Previous studies have made significant contributions to management practices in the power and energy sector. However, current research in power systems still lacks effective utilization of equipment maintenance data. This gap underscores the necessity of developing specialized NLP methods to bridge unstructured textual data with sensor-based analytics in power equipment management.

### 2.2. Named Entity Recognition Methods

In the field of named entity recognition, the BiLSTM-CRF model proposed by Huang et al. [7] opened a new chapter in solving NER problems using deep learning methods. Its introduction made models more concise and robust, becoming a benchmark for addressing NER problems with deep learning. The advent of pre-trained models, including Transformer-based architectures such as BERT, RoBERTa, and XLNet, has revolutionized natural language processing. These models excel at capturing long-term dependencies and hierarchical relationships, providing rich knowledge beneficial for downstream tasks. Consequently, pre-trained models have become instrumental in character embedding and feature extraction. Zhang et al. [21] proposed a novel architecture combining adapted BERT mechanisms with pointer-based identification. Their solution excels at detecting complex entity relationships in Chinese text, particularly when dealing with embedded structures and boundary recognition challenges. Yan et al. [22] proposed TENER, an adaptive Transformer encoder-based architecture for NER designed to model both character-level and word-level features. Chinese named entity recognition presents greater challenges than its English counterpart due to the absence of explicit word boundaries and increased ambiguity [23]. Liu et al. [24] addressed this complexity by introducing USAF, a multimodal Chinese NER method. This approach combines synthetic acoustic features with textual features, employing a multi-head attention mechanism to fuse information from both modalities, resulting in consistent performance improvements.

In recent years, natural language processing has been utilized across various engineering domains. This trend is especially evident in named entity recognition tasks based on text data from diverse engineering fields, attracting growing interest from scholars. Li et al. [8] introduced an innovative neural model for NER which leverages machine reading comprehension and lexicon enhancement to identify both flat and nested entities in bridge inspection texts. Zhang et al. [25] proposed a conditional random fields (CRF)-based method for NER in construction documents, encompassing a corpus design process and a CRF model. Liu et al. [9], leveraging convolutional neural networks, developed a supervised deep learning model for Chinese text classification, generating a corpus solely containing railway faults caused by electromagnetic interference. Subsequently, NER models were constructed using BiLSTM and BERT algorithms.

However, research on NER in the power industry remains limited, especially for maintenance work orders with high complexity of power equipment. In such application scenarios, existing NER methods either depend on external structured resources (e.g., lexicons) or fail to model positional and semantic similarities in noisy textual contexts. These limitations have motivated us to develop a novel NER framework specifically tailored to capture domain-specific entity patterns in power systems while addressing the aforementioned constraints.

### 2.3. Core Components of Transformer

Since the introduction of the Transformer model by Vaswani et al. [26], it has become a foundational architecture in the field of NLP, demonstrating exceptional performance across various tasks [27,28,29]. The Transformer model, with its design entirely based on attention mechanisms, overcomes the limitations of traditional RNN and CNN models in handling long-range dependencies and parallel processing [30]. In recent years, the development and application of the Transformer model have expanded across different domains and tasks, including text generation [31], text classification [32], and speech processing [33] in NLP, as well as image classification [34], object detection [35], and image generation [36] in computer vision. Here, we will introduce several core components of the Transformer model.

#### 2.3.1. Self-Attention

Self-attention is critical in the Transformer model, with its core concept being that each element in a sequence can interact with every other element and adjust its representation based on these interactions. This mechanism effectively captures long-range dependencies between elements within a sequence. Given an input sequence $X=[x_{1},x_{2},\ldots ,x_{n}]$, each element of this sequence undergoes three distinct linear transformations to generate the Query (**Q**), Key (**K**), and Value (**V**):

(1) $$Q,K,V=XW_{q},XW_{k},XW_{v},$$

where $W_{q}$, $W_{k}$, and $W_{v}$ are three learnable weight matrices. After that, the attention score is calculated by:

(2) $$\mathrm{Attn}\left(Q,K,V\right)=\mathrm{softmax}\left(\frac{QK^{T}}{\sqrt{d_{k}}}\right)V,$$

where $d_{k}$ is the dimension of the Key vector.

To enhance the representational capacity of the model, the Transformer architecture incorporates a multi-head attention mechanism. This mechanism performs parallel computations of several independent self-attention layers and concatenates their results, followed by a linear transformation:

(3) $$\mathrm{MultiHead}\left(Q,K,V\right)=\mathrm{Concat}\left({\mathrm{head}}_{1},\ldots ,{\mathrm{head}}_{h}\right)W_{O}.$$

The computation for each head is identical to that of single-head attention, with $W_{O}$ representing the linear transformation matrix for the output.

#### 2.3.2. Linear Attention

Linear attention is an improved method based on self-attention and is designed to reduce computational complexity and memory usage, thereby enhancing efficiency in processing long sequences [37,38]. By redesigning the attention computation process, linear attention decomposes the calculation into several linear operations, allowing optimization through matrix computations [39]. Initially, it maps the Query and Key to a new space by using a specific feature transformation function ϕ:

(4) $$\phi \left(Q\right)=\mathrm{activation}\left(XW_{q}\right),\;\phi \left(K\right)=\mathrm{activation}\left(XW_{k}\right),$$

where the activation can be any nonlinear function such as ReLU or Gaussian kernel [40,41]. Subsequently, the calculation of the attention scores can be expressed as follows:

(5) $$\mathrm{Attn}\left(Q,K,V\right)=\frac{\phi \left(Q\right)(\phi {\left(K\right)}^{T}\cdot V)}{\phi \left(Q\right)\phi \left(K^{T}\right)}.$$

By comparing the computation processes of (2) and (5), it is evident that the computational complexity of attention score calculation decreases from $O(n^{2})$ to O(n). This reduction is achieved because only linear transformations and a few matrix multiplications are required.

#### 2.3.3. Position Embedding

In the Transformer model, elements of the input sequence are processed simultaneously, lacking inherent sequential dependency [42]. While this improves computational efficiency, it also prevents the model from directly perceiving the order of the input data. To address this, position embedding is introduced to explicitly provide positional information, enabling the model to leverage this information to differentiate and understand the sequential structure of the data [26]. This approach maps each word’s position to a fixed-length vector, where each dimension corresponds to a sine or cosine function. Specifically, for a word at position pos, its position embedding vector is given by

(6) $$PE_{\left(pos,2i\right)}=\sin\left(\frac{pos}{10000^{2i/d}}\right),$$

(7) $$PE_{\left(pos,2i+1\right)}=\cos\left(\frac{pos}{10000^{2i/d}}\right),$$

where *d* is the dimensionality of the position embedding vector, and *i* is the index of the position embedding vector’s dimension.

The sinusoidal position embedding employs a combination of sine and cosine functions with geometrically increasing wavelengths. Each dimension *i* of the position embedding corresponds to a unique frequency, enabling the model to capture both fine-grained (high-frequency) and coarse-grained (low-frequency) positional relationships. By interleaving sine and cosine values, the embedding ensures that the positional information for any offset can be represented as a linear function of the original position, thereby facilitating the model’s ability to generalize to unseen sequence lengths. This design inherently encodes absolute positional information but lacks explicit directional awareness between tokens, as we demonstrate in Section 3.2.1.

## 3. Proposed Method

**Figure 2:**
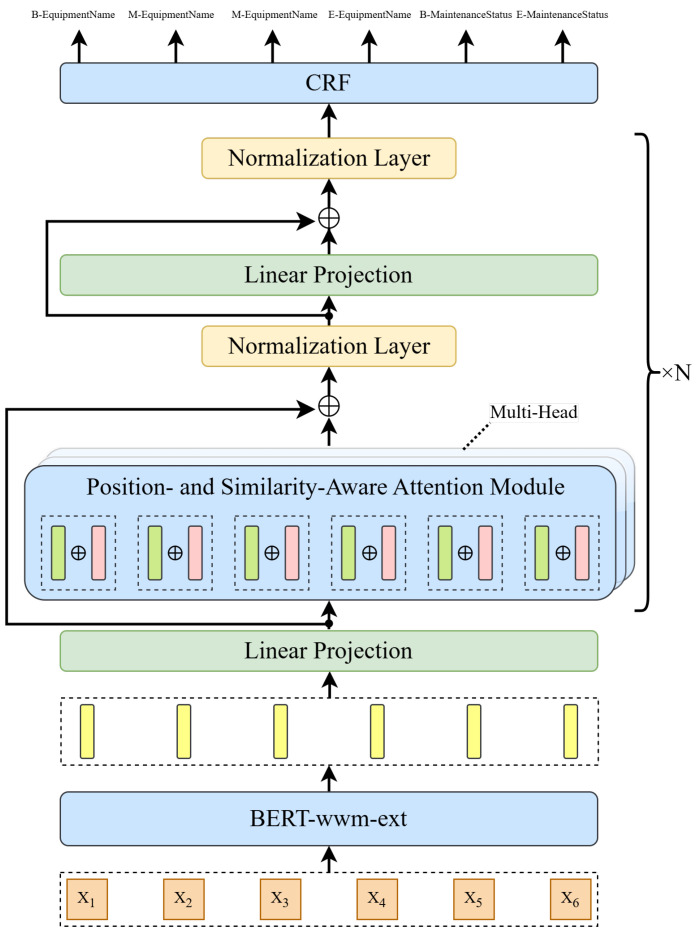
The overall framework of our proposed model.

The overall framework of the proposed model is shown in Figure 2. It mainly consists of three modules: the character embedding module, the position- and similarity-aware attention module, and the CRF module. The character embedding module based on BERT-wwm-ext can generate context-sensitive character vectors, effectively capturing the semantic information of domain-specific terminology in the power industry. The position- and similarity-aware attention module represents the primary innovation of our model. By introducing relative position vectors and vector angle similarity calculations, we have improved the traditional attention mechanism, thereby enhancing the model’s ability to understand context. The position-aware better captures the semantic differences and spatial relationships between different positions, improving the handling of long-distance dependencies; similarity-aware, on the other hand, improves semantic similarity detection by incorporating vector angles, thereby enhancing the model’s ability to recognize semantically related entities at different positions. Furthermore, these improvements allow the model to maintain computational efficiency while reducing computational complexity and improving recognition accuracy. The CRF module optimizes the global consistency of the label sequence through state transition constraints. We will introduce each module in detail in the following subsections.

### 3.1. Character Embedding

In NER tasks, character embedding plays a crucial role. Character embedding transforms words from discrete symbolic forms into continuous vector space representations, mapping the high-dimensional word space to a low-dimensional continuous vector space [43]. On the other hand, character embedding can capture the semantic information of words in the textual context, allowing the model to better understand the textual environment of the entity recognition task [44].

Many NER models use Word2Vec or CNN to complete this process, but these methods have some limitations [45,46]. They are not very good at capturing long-distance dependencies in character sequences because they are usually trained based on the context of local windows, thus losing some important sequence information [47]. BERT has solved the above problems very well. Due to its outstanding performance, it has gradually become the mainstream method for character embedding [48].

We utilize BERT-wwm-ext [49] to perform character embedding on input sequences. BERT-wwm-ext is an enhanced version of BERT, a pre-training model specifically tailored to Chinese corpora. This model is composed of 12 Transformer layers, each containing 768 hidden units and 12 attention heads.

### 3.2. Position- and Similarity-Aware Attention Module

**Figure 3:**
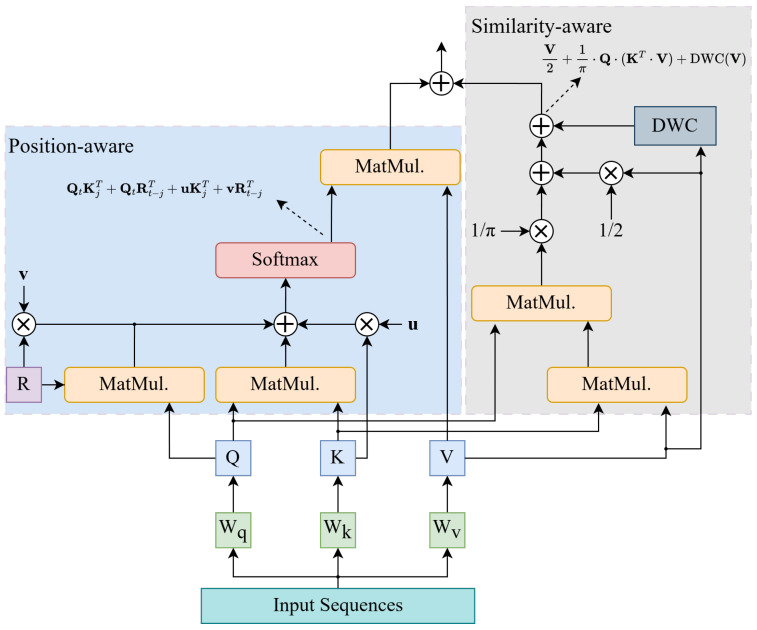
Detailed architecture of position- and similarity-aware attention.

In the NER task, the model needs to recognize the correct entity types and boundaries, which requires that the model have excellent location-awareness and semantic relevance recognition capabilities. Therefore, the original Transformer encoder does not perform the NER task with high accuracy. In this paper, we propose an improved attention mechanism with excellent contextual location awareness and similarity computation capability. Figure 3 illustrates the detailed architecture of this attention mechanism, with the left half presenting position-aware attention and the right half presenting similarity-aware attention.

#### 3.2.1. Position-Aware

During the computation of the original Transformer encoder, a unique embedding is generated for each position through a linear combination of sine and cosine functions [26]. This position embedding gives the model distance awareness but lacks directionality. However, with subsequent self-attention calculations, distance perception becomes weaker [50], as we demonstrate next. First, the position embedding corresponding to the position pos can be derived from (6) and (7)

(8) $$PE_{pos}=\left[\begin{array}{c} \sin(z_{0}pos)\\ \cos(z_{0}pos)\\ \vdots \\ \sin(z_{\frac{d}{2}-1}pos)\\ \cos(z_{\frac{d}{2}-1}pos) \end{array}\right],$$

where *d* denotes the dimension of the position embedding, and $z_{i}=\frac{1}{10000^{2i/d_{k}}}$. During the subsequent computation of attention scores, the position embeddings of two different positions will undergo the following calculation:

(9) $$\begin{array}{cc} PE_{pos}^{T}PE_{pos-k} & =\sum_{j=0}^{\frac{d}{2}-1}\left[\sin\left(z_{j}pos\right)\sin\left(z_{j}\left(pos-k\right)\right)+\cos\left(z_{j}pos\right)\cos\left(z_{j}\left(pos-k\right)\right)\right]\\ & =\sum_{j=0}^{\frac{d}{2}-1}\cos\left(z_{j}\left(pos-\left(pos-k\right)\right)\right)\\ & =\sum_{j=0}^{\frac{d}{2}-1}\cos\left(z_{j}k\right) \end{array},$$

(10) $$\begin{array}{cc} PE_{pos}^{T}PE_{pos+k} & =\sum_{j=0}^{\frac{d}{2}-1}\left[\sin\left(z_{j}pos\right)\sin\left(z_{j}\left(pos+k\right)\right)+\cos\left(z_{j}pos\right)\cos\left(z_{j}\left(pos+k\right)\right)\right]\\ & =\sum_{j=0}^{\frac{d}{2}-1}\cos\left(z_{j}\left(pos-\left(pos+k\right)\right)\right)\\ & =\sum_{j=0}^{\frac{d}{2}-1}\cos\left(-z_{j}k\right) \end{array}.$$

Equations (9) and (10) represent the computation process of position embedding at the current position and position embedding at two different positions before and after. Further, according to the property of cosine function cos(x)=cos(−x), the following can be obtained:

(11) $$PE_{pos}^{T}PE_{pos-k}=PE_{pos}^{T}PE_{pos+k}.$$

It can be seen that it is difficult for the model to directly distinguish the positional relationship between tokens in the process of calculating the attention score. Therefore, in order to make the model contextually position aware with the inclusion of distance and direction, we propose the following approach to obtain the attention scores and position embedding. First, obtain the Query (**Q**), Key (**K**), and Value (**V**),

(12) $$Q,K,V=XW_{q},{\mathrm{XW}}_{k},{\mathrm{XW}}_{v},$$

where **Q**, **K**, **V**$\in R^{l\times d_{k}}$ and $W_{q},W_{k},W_{v}\in R^{d\times d_{k}}$ are the learnable matrices used to compute **Q**, **K**, and **V**, respectively. Next, obtain the relative position information between tokens:

(13) $$R_{t-j}={\left[\ldots \sin\left(\frac{t-j}{10000^{2i/d_{k}}}\right)\cos\left(\frac{t-j}{10000^{2i/d_{k}}}\right)\ldots \right]}^{T},$$

where $R_{t-j}\in R^{d_{k}}$, *t* is the index of the current token, *j* is the index of the adjacent token, and *i* ranges from 0 to $\frac{d_{k}}{2}$. This leads to the position embedding $R_{t}$ and $R_{-t}$ for the forward shift *t* and the backward shift −t, respectively, as

(14) $$R_{t},R_{-t}=\left[\begin{array}{c} \sin(z_{0}t)\\ \cos(z_{0}t)\\ \vdots \\ \sin(z_{\frac{d}{2}-1}t)\\ \cos(z_{\frac{d}{2}-1}t) \end{array}\right],\left[\begin{array}{c} -\sin(z_{0}t)\\ \cos(z_{0}t)\\ \vdots \\ -\sin(z_{\frac{d}{2}-1}t)\\ \cos(z_{\frac{d}{2}-1}t) \end{array}\right].$$

For the forward shift *t*, the sine term is $\sin(z_{0}t)$, and for the backward shift −t, the sine term is $-\sin(z_{0}t)$. This difference in sine allows the model to distinguish between the “preceding” and “succeeding” contexts. Moreover, the values of the sine and cosine terms vary with *t*, reflecting the distance between positions. This position embedding scheme breaks the symmetry in traditional Transformer sine-based position embedding (i.e., $R_{t}=R_{-t}$), enabling the model to directly distinguish directionality.

Then, compute the attention scores:

(15) $$M_{t,j}=Q_{t}K_{j}^{T}+Q_{t}R_{t-j}^{T}+uK_{j}^{T}+vR_{t-j}^{T},$$

(16) $${\mathrm{Attn}}_{p}=\mathrm{softmax}\left(M\right)V.$$

In calculating the attention score according to (15) and (16), the position vectors are calculated separately from the word vectors, and the relevant bias is added. $Q_{t}K_{j}^{T}$ is the attention score between the two tokens at positions *t* and *j* and is calculated using the dot product of the Query vector $Q_{t}$ for token *t* and the Key vector $K_{j}$ for token *j*. This term represents how relevant or compatible the two tokens are, indicating how much attention token *t* should pay to token *j* in the context of the task at hand.

The term $Q_{t}R_{t-j}^{T}$ is a bias term introduced to account for the relative distance between the token at position *t* and the token at position *j*. Here, $R_{t-j}$ is the relative positional embedding between the two tokens. This term helps the model understand the importance of the relative positions of the tokens.

The bias term $u^{T}K_{j}$ incorporates information from the Key vector of token *j*. The learnable parameter u adjusts the attention based on the intrinsic properties of token *j*. This bias term allows the model to emphasize or suppress certain tokens based on their individual characteristics, such as being part of a specific entity or having certain syntactic roles, which is useful in capturing subtle patterns in language.

Similarly, $v^{T}R_{t-j}$ is a bias term that specifically modulates the attention score based on the relative positions of tokens *t* and *j*. The learnable parameter v adjusts the attention based on the direction and distance between the two tokens, further enhancing the model’s ability to capture spatial and sequential relationships.

#### 3.2.2. Similarity-Aware

To perceive more information while minimizing computational cost, we adopt the concept of the linear attention mechanism. The original linear attention mechanism’s drawback is its failure to consider semantic relationships between different positions, relying solely on relative distances between positions [51]. The work conducted in Section 3.2.1 has addressed this deficiency. However, the original linear attention mechanism still suffers from the issue of inferior performance compared to softmax-based attention [52]. Therefore, we introduce the factor of vector angle to enable the model to better perceive similarity information, thereby enhancing its performance.

Define the angle between two vectors as

(17) $$\theta \left(v_{i},v_{j}\right)=\arccos\left(\frac{v_{i}\cdot v_{j}}{∥v_{i}∥\cdot ∥v_{j}∥}\right),$$

where $∥\cdot ∥$ denotes the modulus of the vector. The angle between two vectors is in the range [0,π]. Further, according to (13), we define the similarity between Query and Key vectors as

(18) $$S\left(Q_{i},K_{j}\right)=1-\frac{1}{\pi }\cdot \theta \left(Q_{i},K_{j}\right).$$

It can be seen from the formula that when the similarity between $Q_{i}$ and $K_{j}$ is high, θ tends to 0, then $S(Q_{i},K_{j})$ tends to 1. Conversely, when the similarity between $Q_{i}$ and $K_{j}$ is low, θ tends to π, then $S(Q_{i},K_{j})$ tends to 0. In summary, the function can better reflect the correlation between two vectors.

According to (17), $\theta \left(Q_{i},K_{j}\right)$ can be expressed as

(19) $$\theta \left(Q_{i},K_{j}\right)=\arccos\left(Q_{i}\cdot K_{j}\right).$$

Thus, the similarity formula can be rewritten as

(20) $$S\left(Q_{i},K_{j}\right)=1-\frac{1}{\pi }\arccos\left(Q_{i}\cdot K_{j}\right).$$

Utilizing the relationship between arccos and arcsin:

(21) $$S\left(Q_{i},K_{j}\right)=1-\frac{1}{\pi }\left(\frac{\pi }{2}-\arcsin\left(Q_{i}\cdot K_{j}\right)\right).$$

In order to simplify the computational process and reduce the computational complexity while retaining the accuracy of the similarity measure through approximation, we further transform (21) into using Taylor series expansions,

(22) $$S\left(Q_{i},K_{j}\right)=\frac{1}{2}+\frac{1}{\pi }\left(Q_{i},K_{j}\right)+\frac{1}{\pi }\sum_{k=1}^{\infty }\frac{(2k)!}{2^{2k}{\left(k!\right)}^{2}\left(2k+1\right)}{\left(Q_{i},K_{j}\right)}^{2k+1},$$

where $\frac{1}{2}+\frac{1}{\pi }\left(Q_{i}\cdot K_{j}^{T}\right)$ is a linear similarity-aware term, and the remainder is a higher-order term. The linear similarity-aware term can be directly utilized for computing attention scores, while the higher-order term has a higher complexity and requires further processing. The higher-order term is essentially a nonlinear function that can be approximated by a neural network [53]. Hence, we adopt a depthwise convolution (DWC) module to approximate this computation. The convolutional kernels within DWC are trainable, and through training, these kernels can be adjusted to approximate the effect of the higher-order term within a local scope [52,54]. Additionally, the position-aware segment of the attention module complements long-distance information between non-adjacent tokens. The integration of these two segments enables a more comprehensive approximation of higher-order terms.

Then, the proposed method for computing similarity-aware attention scores is as follows:

(23) $$\begin{array}{cc} {\mathrm{Attn}}_{s} & =S(Q,K)\cdot V\\ \; & \approx \frac{V}{2}+\frac{1}{\pi }\cdot Q\cdot \left(K^{T}\cdot V\right)+\mathrm{DWC}\left(V\right) \end{array}$$

To efficiently approximate the nonlinear higher-order terms, we employ a DWC layer. The DWC applies a set of learnable convolutional kernels ($W_{DWC}$) to the input sequence, enabling local nonlinear transformations. This design aligns with the observation that higher-order interactions in natural language often exhibit locality (e.g., adjacent tokens in a sentence are more likely to interact). Formally,

(24) $$\mathrm{DWC}\left(V\right)=W_{\mathrm{DWC}}\;*V,$$

where ∗ denotes depthwise convolution. During training, $W_{DWC}$ is optimized to capture patterns equivalent to the higher-order Taylor terms. This allows the model to approximate complex nonlinear relationships without explicitly computing high-degree polynomials, significantly reducing computational complexity. The DWC acts as a flexible nonlinear projector. By sliding over the sequence, it mimics the local interactions described by the higher-order Taylor terms.

In conjunction with (16), the computation result of the entire position- and similarity-aware attention module is as follows:

(25) $$\begin{array}{cc} \mathrm{Attn} & ={\mathrm{Attn}}_{p}+{\mathrm{Attn}}_{s} \end{array}$$

### 3.3. CRF

Conditional random field (CRF) is a class of statistical modeling methods often used for structured prediction [55]. It is particularly effective in scenarios where the prediction involves output variables that are interdependent, such as NER tasks. Given a sequence of inputs **X** = $\left[x_{1},x_{2},x_{3},\ldots ,x_{n}\right]$ and a corresponding label sequence **Y** = $\left[y_{1},y_{2},y_{3},\ldots ,y_{n}\right]$, the CRF defines a conditional probability distribution P(Y|X) computed by the following equation:

(26) $$P\left(Y|X\right)=\frac{e^{\sum_{i=0}^{n}A_{y_{i},y_{i+1}}+\sum_{i=0}^{n}P_{i,y_{i}}}}{\sum_{\tilde{Y}\in Y_{X}}e^{\sum_{i=0}^{n}A_{{\tilde{y}}_{i},{\tilde{y}}_{i+1}}+\sum_{i=0}^{n}P_{i,{\tilde{y}}_{i}}}},$$

where the numerator term denotes the exponent of the scoring function given the input sequence **X** and the label sequence **Y**, The denominator term is the sum of the indices of the scoring functions of all possible labeled sequences, ensuring that the probabilities sum to one. $A_{y_{i},y_{i+1}}$ denotes the transfer score for a transfer from state $y_{i}$ to state $y_{i+1}$; $P_{i,y_{i}}$ is the score of the state eigenfunction, denoting the score of the label $y_{i}$ at position *i*, and $Y_{X}$ denotes the set of all possible label sequences. The training objective of the model is to maximize P(Y|X).

## 4. Experiments

### 4.1. Datasets and Experimental Settings

#### 4.1.1. PE-MWO Dataset

The State Grid’s power production management system (PMS) has accumulated a large number of maintenance work orders for power equipment. The text on these work orders is unstructured, primarily consisting of records of various equipment’s maintenance and repair activities during daily operations. The maintenance work order texts have the following three unique characteristics:

- The text length of different work orders varies significantly. In our collected data, the shortest text is 9 characters, while the longest text is 362 characters.
- Since maintenance work orders are written by personnel with different writing habits, the writing format of different work orders varies, and there are differences in the descriptions of the same issue.
- The maintenance work order texts involve a wide variety of entities, specialized vocabulary, abbreviated vocabulary, semantic complexity, and unclear segmentation boundaries.

We first collected raw maintenance work order data from the PMS. Then, we performed data cleaning to remove invalid data, followed by data annotation. We used the BMES annotation system to label the data, where B (Begin) indicates the start of an entity, M (Middle) indicates the middle part of an entity, E (End) indicates the end of an entity, and S (Single) indicates a single-character entity.

After completing data preprocessing and annotation, we constructed the power equipment maintenance work orders (PE-MWO) dataset. This dataset contains extensive maintenance data for various typical power equipment, with a total of 7415 sentences covering 238,869 characters. It involves seven categories of power terminology entities, specifically including *EquipmentName*, *VoltageLevel*, *Line*, *Substation*, *DamagePart*, *MaintenanceStatus*, and *Time*. The meaning and quantity of each entity category are shown in Table 1.

#### 4.1.2. Public Datasets

We also used five public datasets, MSRA [56], Resume [57], OntoNote 4.0 [58], China People’s Daily corpus, and CoNLL-2003 [59], to validate the performance of our proposed model. Table 2 shows the details of each dataset.

- MSRA is a dataset released by Microsoft Research Asia, widely used in the field of natural language processing. This dataset contains a large amount of Chinese text from news reports annotated with named entities, including entity types such as person names, location names, and organization names.
- Resume is specifically designed for NER tasks. It contains a large number of resume texts from various industries and fields, covering a wide range of professions and positions. The dataset includes a total of eight entity categories: country, educational background, location, name, organization, profession, ethnicity, and title.
- OntoNotes 4.0 is a multilingual, multi-domain dataset widely used for natural language processing tasks, covering various domains such as news, conversations, and the web. In this experiment, we selected the Chinese NER portion of the dataset, which includes four entity categories: person, organization, location, and geopolitical entity.
- China People’s Daily corpus, derived from China’s authoritative media outlets, is characterized by its standardized linguistic patterns and formal rhetorical conventions. This comprehensive dataset encompasses multiple domains, including political affairs, economic developments, cultural matters, and societal issues, constituting an extensive repository of domain-specific terminology and nomenclature that exemplifies the practical challenges in named entity recognition research.
- CoNLL-2003 is a benchmark for named entity recognition (NER) and contains English news articles annotated with four entity types: PER (person), LOC (location), ORG (organization), and MISC (miscellaneous). It includes labeled training, development, and test sets, all of which are formatted with word-level BIO tagging. English data derives from the Reuters corpus, supporting model training and evaluation in NLP tasks.

#### 4.1.3. Evaluation Metrics

In our experiments, we used the three evaluation metrics precision (P), recall (R), and F1 score to verify the performance of the model. The specific calculation formulas are as follows:

(27) $$P=\frac{\mathrm{TP}}{\mathrm{TP}+\mathrm{FP}}\times 100\%,$$

where TP denotes the number of samples correctly predicted as positive, FP represents the number of samples incorrectly predicted as positive. Precision (P) indicates the proportion of true positive samples among those predicted as positive by the model. It reflects the model’s accuracy in predicting positive instances.

(28) $$R=\frac{\mathrm{TP}}{\mathrm{TP}+\mathrm{FN}}\times 100\%,$$

where FN represents the number of positive samples incorrectly predicted as negative. Recall (R) indicates the proportion of true positive samples among all actual positive samples that the model correctly identifies. It reflects the model’s coverage of positive instances.

(29) $$F1=2\times \frac{P\times R}{P+R}\times 100\%,$$

F1 score is the harmonic mean of precision and recall designed to balance these two metrics. It provides a trade-off between precision and recall, offering a single measure that accounts for both.

#### 4.1.4. Hyperparameter Settings and Computational Resources

Hyperparameter selection was based on grid search and validation set performance optimization. Specifically, the learning rate was searched within 0.0003, 0.0005, 0.0005, 0.0009, the batch size was adjusted within 16, 32, 64, 128, and the dropout rate was tested within 0.3, 0.4, 0.5. The final parameter combination was determined by maximizing the validation set’s F1 score. For the MSRA dataset, due to GPU memory constraints caused by its large data scale, the batch size was reduced to 16 to accommodate single-GPU training. Ultimately, the hyperparameter configurations employed in model training are detailed in Table 3.

The experiments were conducted on a hardware platform equipped with an NVIDIA GeForce RTX 4090 GPU (NVIDIA, Santa Clara, CA, USA) and an Intel Core i9-14900KF CPU (Intel, Santa Clara, CA, USA) and running Windows 10 Pro. The deep learning framework PyTorch (Version 2.1.2) with CUDA (Version 12.1) acceleration was utilized under a Python (Version 3.10) environment managed by Anaconda, as shown in Table 4.

### 4.2. Ablation Study

To validate the effectiveness of our proposed improvements, we conducted an ablation study on the PE-MWO dataset. The results are presented in Table 5.

In Table 5, the baseline model is configured as Transformer+CRF. When only the position-aware function was applied, the F1 score improved by +4.3% (from 76.4% to 80.7%) compared to the baseline. The position-aware function enhances the model’s ability to capture the relative distance and direction between tokens, which is crucial for tasks like NER. By incorporating relative positional embeddings and learnable bias terms, the mechanism adjusts the attention scores based on both token relevance and their spatial relationships. This makes the model more sensitive to the proximity and direction of tokens, improving its ability to distinguish entity boundaries and relationships.

When only the similarity-aware function was applied, the F1 score increased by +3.2% (from 76.4% to 79.6%) compared to the baseline. The similarity-aware function introduces vector angle-based semantic relevance, which defines similarity through the angular relationship between the Query and Key vectors and quantifies semantic alignment more accurately than traditional dot product attention. This approach ensures that tokens with high semantic relevance receive stronger attention weights, while irrelevant tokens are suppressed.

**Figure 4:**
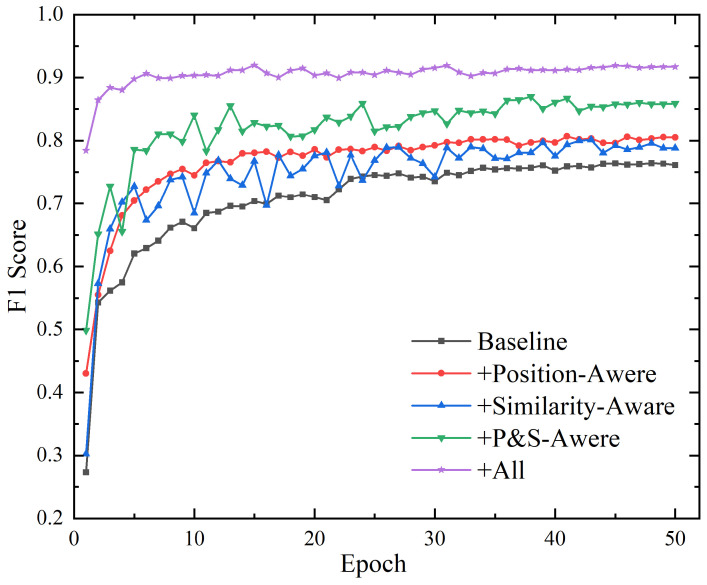
The variation of F1 values for different modules on the PE-MWO dataset.

The joint use of both position-aware and similarity-aware modules achieved an F1 score of 86.9%, surpassing the baseline by +10.5%—exceeding the sum of individual improvements (4.3% + 3.2% = 7.5%). This indicates synergistic interaction rather than additive effects. Specifically, the combination of position-aware and similarity-aware modules enables the model to not only capture the sequential relationships between words but also more accurately identify the semantic correlations between them. The integration of both mechanisms enhances the model’s robustness when dealing with complex texts. The position-aware function ensures that the model remains efficient when handling entities that require strict sequential order, while the similarity-aware function guarantees that semantically related entities, regardless of their positions, are effectively recognized. As shown in Figure 4, the synergistic effect of these modules not only improves performance but also accelerates convergence, demonstrating that the complementary and synergistic interaction between the two modules is both rational and effective.

The full model achieved a state-of-the-art F1 score of 91.8%, outperforming the combined modules by +4.9%. The improvement is attributed to the integration of BERT-wwm-ext with the position- and similarity-aware modules, which further optimize character embeddings and contextual understanding. By incorporating the CRF module, the model is able to refine the prediction of label sequences, enhancing overall recognition accuracy. Ultimately, this full model integrates the strengths of each module, enabling the model to excel in recognizing various entity types, particularly in the complex domain of power equipment maintenance work orders, showcasing its strong adaptability and efficient performance.

Figure 4 illustrates the F1 score variation curves for different combinations on the test set. Overall, our proposed position-aware and similarity-aware enhancements both contribute to performance improvements over the baseline model. Notably, the integration of both awareness functions results in substantial enhancements in both model performance and convergence speed.

In summary, the superior predictive accuracy of our proposed model can be attributed to its ability to precisely capture contextual positional information and semantic relevance. In practical applications, this model can efficiently and accurately identify specialized terminology within maintenance work orders in the power sector.

### 4.3. Visual Analysis

#### 4.3.1. Confusion Matrix Heatmaps

**Figure 5:**
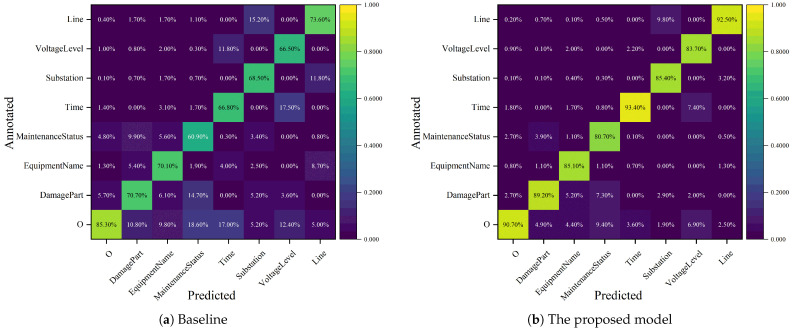
Confusion matrix heatmaps of model predictions.

Figure 5 presents confusion matrix heatmaps constructed from the prediction results of both the baseline and our proposed model, visualizing their performance across different entity categories. The x-axis represents model predictions, while the y-axis indicates the annotated labels in the dataset. The raw values are normalized by column, with color intensity representing the magnitude of the normalized proportions. The diagonal cells display the proportion values, representing the precision of model predictions for each entity category, while other cells indicate the proportions of samples predicted as one category but actually belonging to other entity categories.

From a holistic perspective, the darker-colored regions in the heat map are more concentrated on the diagonal, indicating the improvement in the prediction accuracy and stability of our proposed model for each entity class. Additionally, inter-category confusion is significantly reduced, as evidenced by the generally lighter colors in non-diagonal regions.

Specifically, as depicted in Figure 5a, the baseline model exhibits limited accuracy in recognizing *MaintenanceStatus* entities and demonstrates a tendency to confuse them with *DamagePart* entities. This limitation arises primarily due to the nature of *MaintenanceStatus* entities, which represent specific operational tasks often described by diverse and lengthy textual phrases. On the other hand, *DamagePart* entities are usually shorter and more directly associated with specific components of equipment. The original Transformer model struggles to differentiate these two types of entities because it lacks the ability to effectively capture the contextual relationships between them, especially in instances where *MaintenanceStatus* and *DamagePart* frequently co-occur in the same text.

Our proposed model, however, improves performance in these cases by leveraging position- and similarity-aware attention mechanisms. Specifically, the position-aware component ensures that the model retains strong awareness of the relative positions of tokens, helping it recognize the sequential structure of sentences. This is particularly beneficial when distinguishing between *MaintenanceStatus* and *DamagePart* entities, which often appear in similar contexts but in different positions within the sentence. Additionally, the similarity-aware component of the model enhances its ability to detect semantic similarities between tokens, which allows it to better differentiate between entity types that might share similar vocabulary but have different meanings depending on context. This dual attention mechanism enables the model to understand both semantic relevance and structural position, thereby reducing errors in recognizing and classifying these entities.

Further error analysis reveals that the baseline model tends to confuse *VoltageLevel* with *Time* entities, likely due to their similar numeric forms. However, the model’s understanding of context improves significantly with our approach. The similarity-aware attention mechanism helps the model recognize that, although both entities may contain numeric values, their roles within the sentence differ based on surrounding words and the overall context of the sentence. For instance, *VoltageLevel* typically appears in contexts describing equipment specifications, while *Time* is often associated with maintenance actions or schedules. Our model is able to better differentiate these by considering the similarity between tokens in the context of the entire sentence, rather than just relying on surface-level features such as numeric form.

Although our proposed model improves on the baseline model in all entity categories, certain entity categories are still more difficult to accurately recognize for the following reasons:

- Long-distance dependency problems: Although the model is able to capture contextual information, there may still be the issue that it cannot fully capture long-distance dependencies for some entity categories, especially those spanning longer sentences. This is particularly important for entity classes such as *DamagePart* and *MaintenanceStatus*, whose descriptions are often long and may be semantically intertwined with other entities, making it more difficult for the model to capture their relationships.
- The problem of polysemy and synonymy: In the context of power equipment maintenance, certain words are polysemous and may share vocabulary with multiple entity categories. For example, *Substation* may have some semantic overlap with *Line* or *EquipmentName*, leading to unstable model categorization between these categories. Even though the model mitigates this problem to some extent through location and similarity-aware mechanisms, the model may still produce misclassification in some cases due to the lack of deeper semantic understanding.
- High-dimensional input features: Although BERT-wwm-ext is able to provide effective feature representation for Chinese text, when the dimensionality of the input features is too high (especially in long text or complex entity descriptions), the model may capture too many irrelevant features, which in turn affects the prediction accuracy.

To further improve the model’s performance on these categories, several strategies could be explored. Data augmentation techniques, such as random noise injection or synthetic data generation, may help alleviate class imbalance and increase the representation of less-frequent entity types. Additionally, refining the model’s ability to capture long-range dependencies could further enhance its contextual understanding, particularly for complex entities. Incorporating multi-task learning or transfer learning approaches could also strengthen the model’s robustness. These strategies will contribute to improving the model’s overall accuracy and its ability to distinguish between similar entity types.

#### 4.3.2. Comparative Analysis of Prediction Instances

**Figure 6:**
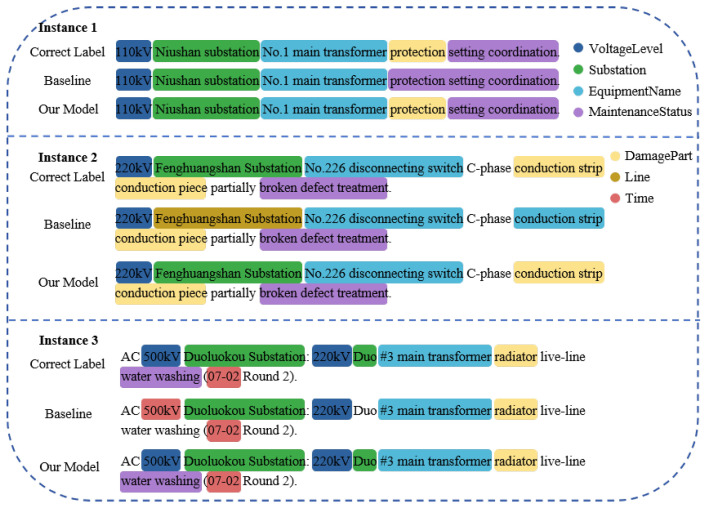
Comparison of predictions between the baseline and the proposed model (original Chinese texts are translated into English for clarity).

To provide an intuitive demonstration of the improvements achieved by our proposed model over the baseline, we present several representative prediction instances in Figure 6 for comparative analysis. These examples encompass texts with varying sentence lengths and writing styles. The three sentences are as follows: “110 kV Niushan substation No.1 main transformer protection setting coordination”; “220 kV Fenghuangshan Substation No.226 disconnecting switch C-phase conduction strip conduction piece partially broken defect treatment”; and “AC 500 kV Duoluokou Substation: 220 kV Duo #3 main transformer radiator live-line water washing (07-02 Round 2)”.

In Instance 1, the baseline model incorrectly identified *DamagePart* as part of *MaintenanceStatus*. In Instance 2, the baseline model confused *Substation* with *Line* and misclassified *DamagePart* as *EquipmentName*. In Instance 3, the baseline model incorrectly identified *VoltageLevel* as *Time*, which also has a numeric form, and failed to recognize some entities. In contrast, the proposed model successfully avoided these errors, demonstrating a superior ability to distinguish between semantically similar entity categories. This indicates that the proposed model can accurately capture the positional information of entities within a sentence and effectively understand the dependency relationships between entities.

### 4.4. Overall Performance Comparison

In this section, we conduct a comprehensive comparison of our proposed model with other models on five public datasets: MSRA, OntoNotes 4.0, Resume, the China People’s Daily corpus, and CoNLL-2003. The F1 score is used as the evaluation metric for model performance.

Table 6, Table 7, Table 8, Table 9 and Table 10 present the performance of our model alongside several advanced Chinese named entity recognition models across five public datasets: MSRA, OntoNotes 4.0, Resume, the China People’s Daily corpus, and CoNLL-2003. Evidently, our model consistently outperforms all other models in overall performance across all datasets.

From the perspective of dataset size, our model demonstrates robust performance on both larger datasets such as MSRA, OntoNotes 4.0, and the China People’s Daily corpus, as well as smaller datasets like Resume. This indicates that our model is capable of rapidly capturing contextual information from training texts and exhibits greater robustness to the quantity of training samples compared to other models.

From the perspective of dataset domain coverage, MSRA and the China People’s Daily corpus primarily consist of news report texts, while Resume predominantly contains highly specialized resume texts. Our model demonstrates superior performance over other advanced models, both in news texts with long sentences and complex syntactic structures and in resume texts containing a large number of abbreviations and specialized terms. The experimental results clearly validate the reliability of our designed model for Chinese named entity recognition across various domains.

From the perspective of language adaptability, our model, originally designed for Chinese, shows promising performance on the English CoNLL-2003 dataset. The position- and similarity-aware attention mechanism, which is agnostic to language morphology, allows the model to effectively capture contextual and semantic relationships in English text, even with its distinct syntactic structures and word boundaries. Experimental results confirm that our model maintains competitive performance on this English dataset, demonstrating its versatility beyond Chinese-specific challenges.

## 5. Conclusions and Future Perspectives

Power equipment maintenance work orders serve as a critical data source in power equipment analysis and management. This paper systematically examines the linguistic characteristics of Chinese power domain texts and the technical challenges associated with named entity recognition tasks in this specialized context. In this study, we first construct a domain-specific dataset (PE-MWO) encompassing seven entity categories, comprising 7415 sentences and 238,869 characters, thereby providing a high-quality benchmark resource for both academic research and industrial applications in named entity recognition. Subsequently, we propose a novel named entity recognition model. The primary scientific contribution of this model lies in its innovative position- and similarity-aware attention mechanism module, which synergistically integrates directional positional embedding with vector angular similarity computation. This architectural innovation effectively addresses the limitations of conventional attention mechanisms by explicitly encoding relative positional relationships and semantic associations while maintaining computational efficiency. Experimental validation demonstrates that the proposed model achieves a 15.4% improvement in F1 score over baseline models on the PE-MWO dataset.

Furthermore, the model exhibits strong generalizability across five public datasets (MSRA, Resume, OntoNotes 4.0, the China People’s Daily corpus, and CoNLL-2003), with F1 scores reaching 96.39%, 96.78%, 84.39%, 96.95%, and 93.91%, respectively. This suggests that the model has strong comprehensive performance on texts in multiple languages and in multiple domains. Practically, this research bridges the gap between unstructured maintenance texts and sensor-driven equipment monitoring systems. The automated extraction of entities such as equipment names, damaged components, and maintenance actions facilitates the integration of textual data with sensor measurements, enabling predictive maintenance and lifecycle management. The modular design of the model also supports adaptation to other industrial domains, offering a scalable solution for knowledge graph construction and cross-domain data fusion.

The current work opens up several promising directions for future research:

- Sensor Data Integration: Our structured maintenance information could be integrated with sensor measurements to provide more comprehensive equipment health monitoring. This integration would combine human expert knowledge from maintenance records with quantitative sensor data.
- Sensor-Based Validation: Future research could explore using sensor measurements to validate and enrich the extracted maintenance information, helping to establish more reliable equipment status assessment systems.
- Security Risk Assessment: The extracted structured information could be utilized to develop more sophisticated power equipment security risk assessment models, helping to identify potential vulnerabilities and prevent equipment failures.
- Smart Grid Applications: Future work could focus on incorporating the extracted maintenance information into smart grid management systems, supporting more intelligent decision-making in power equipment operation and maintenance scheduling.

In conclusion, this study provides significant support for the management of power equipment and the construction of smart grids while also offering valuable references for similar text processing tasks in other engineering domains. The proposed method and dataset contribute to advancing the field of power equipment information processing and management, laying a foundation for more intelligent and efficient power system operation.

## Figures and Tables

**Table 1:** The meaning and quantity of each entity category.

Entity Category	Entity Meaning	Entity Quantity
EquipmentName	The names of the equipment in the maintenance work orders	4184
VoltageLevel	The voltage levels of the equipment	2802
Line	The lines to which the equipment belongs	1496
Substation	The substations to which the equipment belongs	1474
DamagePart	The damaged parts of the equipment	2705
MaintenanceStatus	The specific maintenance details of the equipment	4514
Time	The maintenance time of the equipment	228

**Table 2:** Summary statistics of different datasets.

Dataset	Type	Train (k)	Dev (k)	Test (k)	Entity Type
MSRA	Sentences	46.4	4.4	4.4	3
	Chars	2169.9	172.6	172.6	
	Entities	74.8	6.2	6.2	
Resume	Sentences	3.8	0.46	0.48	8
	Chars	124.1	13.9	15.1	
	Entities	13.4	1.5	1.6	
OntoNote 4.0	Sentences	15.7	4.3	4.3	4
	Chars	491.9	200.5	208.1	
	Entities	13.3	6.9	7.7	
People’s Daily	Sentences	20.86	2.32	4.64	3
	Chars	979.2	109.9	219.2	
	Entities	33.9	3.8	7.7	
CoNLL-2003	Sentences	15.0	3.4	3.6	4
	Chars	203.6	51.4	46.4	
	Entities	23.5	5.9	5.6	

**Table 3:** Hyperparameter settings.

Hyperparameters	MSRA	Other Datasets
Number of layers	2	2
Number of head	6	6
Optimizer	Adam	Adam
Learning rate	0.0007	0.0009
Batch size	16	64
Dropout	0.4	0.4
Epochs	50	50

**Table 4:** Computational resources and hardware configuration.

Configuration Item	Specifications/Model
GPU	NVIDIA GeForce RTX 4090
CPU	Intel Core i9-14900KF
Operating System	Windows 10 Pro
Deep Learning Framework	PyTorch 2.1.2 + CUDA 12.1
Python Environment	Python 3.10

**Table 5:** Ablation results of the proposed model.

Model	P (%)	R (%)	F1 (%)
Baseline ^1^	76.9	75.9	76.4
+Position-Aware ^2^	82.9	78.6	80.7
+Similarity-Aware ^3^	76.8	82.6	79.6
+Position- and Similarity-Aware ^4^	84.9	89.0	86.9
+All ^5^	**91.5**	**92.1**	**91.8**

^1^ Transformer+CRF. ^2^ Using only the position-aware function. ^3^ Using only the similarity-aware function. ^4^ Using the full position- and similarity-aware attention. ^5^ BERT-wwm-ext+P&S-Aware+CRF. Bold font represents the highest-performing results.

**Table 6:** Performance comparison of different methods on MSRA.

Model	P (%)	R (%)	F1 (%)
Zhang et al., 2019 [60]	90.59	91.15	90.87
Guo et al., 2020 [61]	89.76	91.82	90.55
Gong et al., 2020 [62]	94.83	93.61	94.22
Li et al., 2022 [63]	95.00	94.96	94.97
Chen et al., 2023 [64]	96.84	93.78	95.29
Ke et al., 2024 [65]	96.28	96.23	96.26
Han et al., 2024 [66]	95.83	**96.84**	96.34
Ours	**97.08**	95.71	**96.39**

Bold font represents the highest-performing results.

**Table 7:** Performance comparison of different methods on OntoNotes 4.0.

Model	P (%)	R (%)	F1 (%)
Gong et al., 2020 [62]	77.77	76.32	77.04
Wang et al., 2022 [67]	-	-	81.87
Wang et al., 2022 [68]	-	-	82.43
Chen et al., 2023 [64]	77.64	76.26	76.94
Tian et al., 2023 [69]	**82.61**	84.29	83.44
Han et al., 2024 [66]	82.16	83.51	82.83
Zhang et al., 2024 [21]	-	-	83.25
Ours	82.59	**86.27**	**84.39**

Bold font represents the highest-performing results.

**Table 8:** Performance comparison of different methods on Resume.

Model	P (%)	R (%)	F1 (%)
Guo et al., 2020 [61]	94.9	94.56	94.62
Wang et al., 2022 [68]	-	-	96.21
Wang et al., 2022 [67]	-	-	96.53
Mai et al., 2022 [70]	**96.91**	96.26	96.58
Chen et al., 2023 [64]	96.14	96.52	96.33
Tian et al., 2023 [69]	96.69	96.81	96.75
Zhang et al., 2024 [21]	-	-	96.23
Ours	96.4	**97.17**	**96.78**

Bold font represents the highest-performing results.

**Table 9:** Performance comparison of different methods on the China People’s Daily corpus.

Model	P (%)	R (%)	F1 (%)
Zhang et al., 2022 [71]	93.23	92.42	92.82
Zhang et al., 2022 [72]	94.71	92.42	93.64
Liu et al., 2024 [73]	92.17	90.63	91.4
Ke et al., 2024 [65]	95.93	**96.45**	96.19
Ours	**97.53**	96.38	**96.95**

Bold font represents the highest-performing results.

**Table 10:** Performance comparison of different methods on CoNLL-2003.

Model	P (%)	R (%)	F1 (%)
Yi et al., 2021 [74]	91.18	91.36	91.27
Chen et al., 2023 [75]	-	-	93.09
Fei et al., 2023 [76]	92.96	**93.85**	93.4
Chang et al., 2023 [77]	-	-	93.46
Yu et al., 2024 [78]	-	-	93.42
Ours	**94.05**	93.76	**93.91**

Bold font represents the highest-performing results.

## Data Availability

The datasets generated and/or analyzed within the current study are available from the corresponding author upon reasonable request. Requests to access the datasets should be directed to qushaocheng@mail.ccnu.edu.cn.
